# Serum sickness following rituximab therapy in a patient with pemphigus vulgaris: A case report

**DOI:** 10.1002/ccr3.3642

**Published:** 2020-12-08

**Authors:** Mohamad Y. Khatib, Solaiman M. Allafi, Abdulqadir J. Nashwan

**Affiliations:** ^1^ Medical Intensive Care Department Hazm Mebaireek General Hospital (HMGH) Hamad Medical Corporation (HMC) Doha Qatar; ^2^ Education & Practice Development Hazm Mebaireek General Hospital (HMGH) Hamad Medical Corporation (HMC) Doha Qatar; ^3^ University of Calgary in Qatar (UCQ) Doha Qatar

**Keywords:** human antichimeric antibodies, immunosuppressant, pemphigus vulgaris, rituximab, serum sickness

## Abstract

Serum sickness, a reaction presenting with a classic triad (fever, rash, myalgia/arthralgia), is considered as a rare adverse event following monoclonal antibodies and specifically following treatment with rituximab. This report describes a case of serum sickness in a newly treated young male patient with rituximab for pemphigus vulgaris.

## Graphical abstract

Clinicians should be vigilant for potential acute or delayed serum sickness as possible adverse reactions to weigh the benefits and risks of rituximab therapy in patients with pemphigus vulgaris.

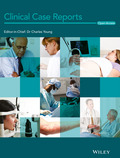

## 1 BACKGROUND

Monoclonal antibodies have been used for the modulation of immune responses in several disorders, including cancer, autoimmune, and infectious diseases.^1^

Rituximab (RTX) is a chimeric monoclonal antibody that specifically binds to the B‐cell surface antigen CD20 which mediates cytotoxicity in these cells.^2^, ^3^, ^4^ The mechanism of action of RTX includes regulating cell‐cycle signaling, inducing apoptosis, and improving cytotoxic drug cells' sensitization, antibody‐dependent cellular cytotoxicity, and complement‐mediated cytotoxicity.^4^

Rituximab is used in the management of previously untreated or treated adults with CD20^+^ chronic lymphocytic leukemia (CLL),^5^ granulomatosis with polyangiitis,^6^ microscopic polyangiitis,^7^ non‐Hodgkin's lymphomas,^8^ pemphigus vulgaris,^9^ and rheumatoid arthritis,^10^ and has other off‐label uses in treating acquired thrombotic thrombocytopenic purpura,^11^ Waldenström's macroglobulinemia,^12^ and refractory autoimmune hemolytic anemia.^13^

As a monotherapy, RTX (1000 mg) is administered weekly for 1 month or every 3‐4 weeks when used in combination with chemotherapy.^14^

The adverse events of RTX might be early (eg, fever, cough, and dyspnea) or late (eg, agranulocytosis or severe infection).^15^

Pemphigus is one of the autoimmune diseases characterized by cutaneous blisters and erosions and mediated by autoantibodies. Pemphigus vulgaris (PV) is the major progressive subtype of pemphigus and may lead to death if it is not treated.^16^

Serum sickness is an uncommon hypersensitivity undesirable reaction due to antigens, characterized by fever, rash, and arthralgia.^17^ RTX is inducing serum sickness by producing human antichimeric antibodies.^18^ Although serum sickness is commonly reported in patients with autoimmune disease, few reports were published for patients with PV.

## 2 CASE PRESENTATION

A 33‐year‐old male patient was admitted with a history of fever, malaise, arthralgia, and myalgia for one day. Two weeks ago, he was started on RTX (1000 mg IV 2 times per week) for the treatment of severe refractory pemphigus vulgaris and he responded very well as per the dermatologist, in addition to his previous treatment with intravenous immunoglobulin (IVIG) (2 g/kg per cycle), azathioprine (2.5 mg/kg), and prednisolone (2 mg/kg per day) which were started earlier for the last 2 years. The symptoms appeared 11 days after the second dose.

Subsequent physical examination revealed fever (a temperature of 38.5°C), hemodynamic instability with sinus tachycardia of 130‐140 bpm, mild tachypnea, malaise, generalized body rash (cutaneous blisters), and generalized body pain. He reported severe pain while moving his elbows, shoulders, knees, wrists, ankles, spine, metacarpophalangeal joints, and temporomandibular joints. However, no effusions, swelling, and erythema were noted. He was immediately started on a wide‐spectrum antibiotic (piperacillin‐tazobactam 4.5 g Q6 hours) at the beginning in view of suspected sepsis and was discontinued shortly after admission.

Subsequent laboratory investigations revealed leukocyte count, 26.4 (4‐10 × 10^3^/uL); neutrophils, 84.3%; platelet count, 267 (150‐400 x 10^3^/uL); erythrocyte count, 6.4 (4.5‐5.5 × 10^6^/uL); C‐reactive protein level, 112 (0.0‐5.0 mg/L); uric acid level, 7.7 (3.4‐7.0 mg/dL); and creatinine, 144 (62‐106 umol/L), and ferritin was normal. Coagulation profile, electrolytes, and C3 and C4 levels were within normal ranges, urinalysis was within normal range except a trace of blood, and blood cultures and viral panel were negative.

Based on the medication history, physical examination, and excluding other potential causes, the patient was initially diagnosed with acute serum sickness associated with RTX therapy. Instantly, he was treated with methylprednisolone of 1 mg/kg/day divided into 2 doses, ample analgesia with paracetamol, morphine, and fentanyl patches. He was immediately started on a wide‐spectrum antibiotic at the beginning in view of suspected sepsis and was discontinued shortly after admission. His signs and symptoms resolved within 2 days from initiation of the abovementioned treatment, and his follow‐up laboratory test results were normal. Four days later, he was discharged home and referred to a dermatology and allergy clinic where the treating physician has discussed with the primary physician the treatment options and possible complications for resuming the patient on RTX.

## 3 DISCUSSION

Rituximab is a novel therapeutic agent for severe and recalcitrant pemphigus vulgaris (PV).^19^ In patients with PV, human antichimeric antibodies (eg, RTX) are known to cause treatment failure and adverse effects, especially with intravenous administration.^20^

Rituximab‐induced serum sickness (RISS) has been reported earlier in various autoimmune disorders including rheumatoid arthritis, Sjogren's syndrome, and hematological malignancies.^21^ Typically, it has been explained by the presence of the murine component in RTX and B‐cell lysis by forming complexes with antibodies due to the delivery of intracellular antigens to the serum which then precipitates systematically in the synovial membranes of joints.^22^, ^23^

A 2015 literature review identified 33 reported cases associated with RTX where most of the reported cases were related to an underlying rheumatologic condition (such as Sjögren's syndrome). The classic triad (fever, rash, and arthralgia) of serum sickness was reported less than half of cases. The mean time from exposure to symptom onset with the RTX first dose was almost double compared with the second dose.^24^

A recent study has described the epidemiological and clinical characteristics of 37 cases of RISS reported in France. Serum sickness occurred mainly 12 days after the first injection (54%). The most frequent manifestations were rheumatologic symptoms (92%), fever (87%), and skin lesions (78%). The incidence was significantly higher when RTX was used for autoimmune diseases than for hematological malignancies.^25^

The role of RTX in severe refractory PV has been studied in the past few years. Some of the documented adverse effects include severe infections such as pneumonia, progressive multifocal leukoencephalopathy, anaphylaxis, and Stevens‐Johnson syndrome.^19^

In our case, the treating physician did not recommend resuming RTX to prevent any further severe reaction. Mainly, the diagnosis of serum sickness depends on clinical features. Other causes such as malignancy and any infection that can trigger serum sickness should be ruled out. In this case, investigations such as blood investigations (eg, CBC, complements C3 and C4, blood culture), ultrasonography abdomen, and urine analysis helped in ruling out malignancy and infectious potential causes. In our patient, clinical presentation, medication history, and quick response to treatment helped in making the diagnosis of RISS. Overall, all the clinical features, laboratory findings, and quick response to corticosteroids were suggestive of serum sickness due to RTX (which was started 2 weeks ago) after excluding other possible causes.

## 4 CONCLUSION

Rituximab is considered an important treatment option in patients with moderate‐to‐severe refractory pemphigus vulgaris. RTX has been shown to be effective and well‐tolerated in several autoimmune conditions. However, there are still concerns about its long‐term adverse effects. Clinicians should be vigilant for potential acute or delayed serum sickness as possible adverse reactions to weigh the risks and benefits of continuing/reintroducing RTX for patients with pemphigus vulgaris. Further studies reporting the adverse effects of RTX are required to establish its safety and tolerability.

## CONFLICT OF INTEREST

The authors declare that they have no competing interests.

## AUTHOR CONTRIBUTIONS

MYK, SMA, and AJN: collected data, conducted literature search, and prepared the manuscript. All authors: read and approved the final manuscript.

## ETHICAL APPROVAL

The article describes a case report. Therefore, no additional permission from our Ethics Committee was required.

## CONSENT FOR PUBLICATION

The consent for publication was obtained.

## Data Availability

All data generated or analyzed during this study are included in this published article.
